# Follicular lymphoma transforming to DLBCL and reverting back to follicular lymphoma at relapse—a case report

**DOI:** 10.1186/s43046-020-00035-3

**Published:** 2020-05-14

**Authors:** M. C. Suresh Babu, Antony George Francis Thottian, D. Lokanatha, Linu Abraham Jacob, K. N. Lokesh, A. H. Rudresha, L. K. Rajeev, Saldanha Smitha, Syed Adil Hassan, Khandare Pravin Ashok, C. S. Premalatha, M. N. Suma

**Affiliations:** 1 Department of Medical Oncology, Kidwai Cancer Institute, Dairy Circle, Bengaluru, 560029 India; 2 Department of Pathology, Kidwai Cancer Institute, Bengaluru, 560029 India

**Keywords:** Follicular lymphoma, DLBCL, Histologic transformation, Case report

## Abstract

**Background:**

Transformation of low-grade follicular lymphoma to high-grade diffuse large B cell lymphoma (DLBCL) is known. However, the opposite is not commonly reported. In this report, we present a case of follicular lymphoma that underwent transformation to DLBCL. Three years after treatment for histologic transformation, the patient presented again with low-grade follicular lymphoma at the same site which is unusual in the natural history of follicular lymphoma.

**Case presentation:**

A 50-year-old female patient presented to us with complaints of slowly progressing swelling in the neck on the left side for a duration of 1 year. Past history of the patient revealed a diagnosis of follicular lymphoma in 2004 for which the patient had taken prednisolone and chlorambucil. Details of staging were not available with the patient. After a complete work-up, she was diagnosed as DLBCL, stage IIIE. She was treated with 6 cycles of CHOP regimen. She had very good response to chemotherapy. However, she defaulted and was lost to follow-up. She presented again after 3 years with history of painless progressive swelling in the right side of the neck for the last 1 year. Examination revealed cervical lymph nodes and ascites. This time, a repeat biopsy and immunohistochemistry was suggestive of follicular lymphoma. In view of significant ascites, she was started on chemotherapy with CVP regimen. After 6 cycles, she has good partial response and resolution of ascites. She is currently on follow-up.

**Conclusions:**

We have presented a case of FL that has transformed to DLBCL after 10 years of diagnosis. After HT, she was treated with CHOP chemotherapy and the patient relapsed again after 3 years with follicular lymphoma histology. This case highlights the unique and varied natural history of follicular lymphoma that may be attributed to different subclones of malignant cells that may have arisen from a common progenitor FL cell and differential effect of chemotherapy on these subclones.

## Background

Follicular lymphoma (FL) is the most commonly diagnosed indolent lymphoma with an incidence ranging from 2.1 to 3.4 per 100,000 population according to different national and international registries and accounting for up to 35% of all non-Hodgkin lymphoma (NHL) diagnosed in western countries [1–4]. It is the malignant counterpart of the normal follicular B cells. FL is a heterogeneous clinicopathologic entity with varying risk factors dictating disease progression and survival. Histologic transformation (HT) of FL is an event during the natural history of the disease in which the lymphoma evolves to an aggressive course with histologic appearance of DLBCL. HT portends a dismal prognosis and is expected to happen in about 1–4% of cases of FL per year [5–7]. However, it is unusual to see a case of FL that has transformed to DLBCL to revert back to the indolent FL after treatment. In this report, we describe a case of FL that transformed to DLBCL 10 years after diagnosis and post-treatment for HT relapsing again as FL.

## Case presentation

**Fig. 1:**
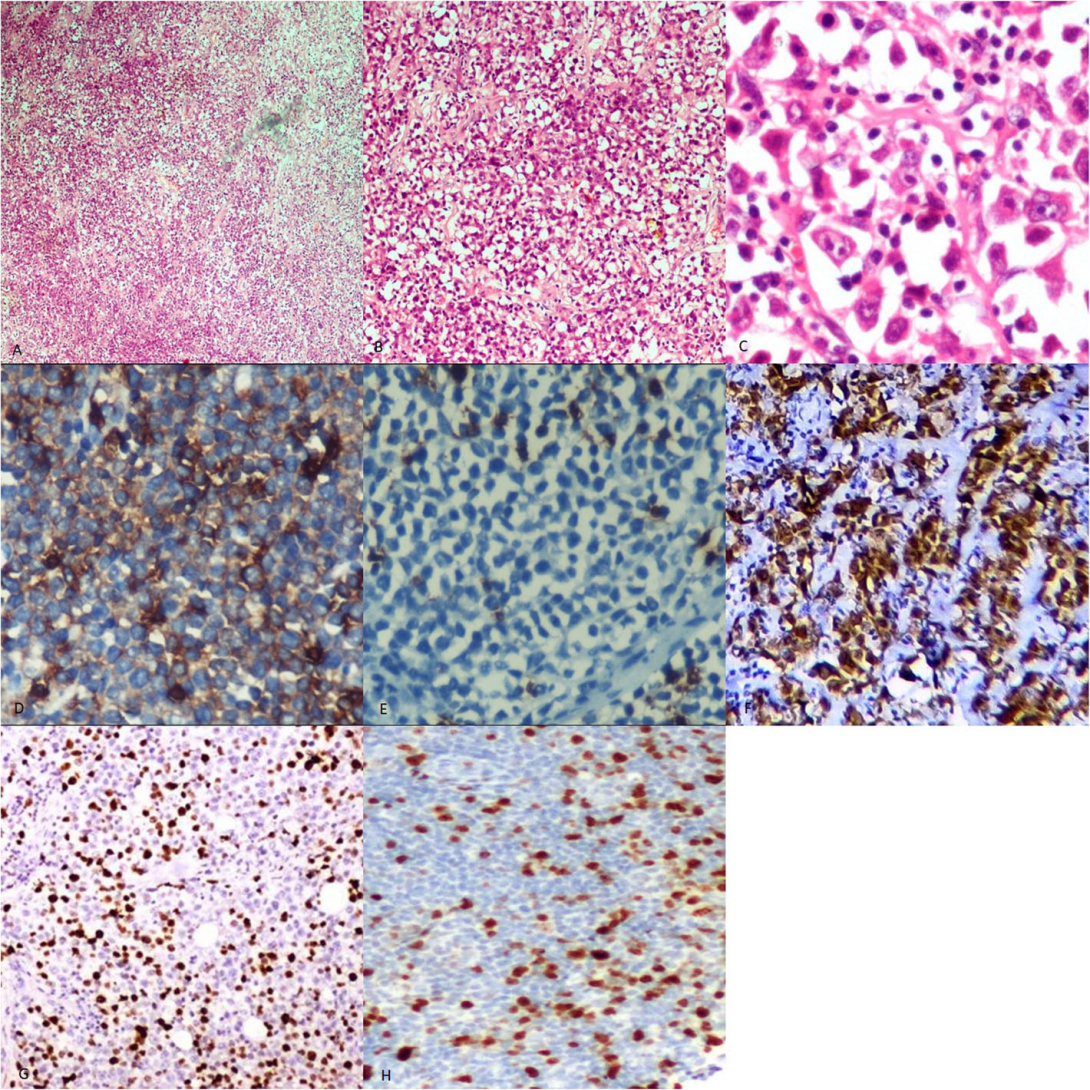
Histopathology Images of Biopsy specimen done in 2015 suggestive of DLBCL. **a** 40× scanner view of the lymph node showing diffuse sheets of lymphoid cells. **b** 100× low-power view displaying atypical lymphoid cells. **c** 400× high-power view displaying large atypical lymphoid cells having vesicular nuclear chromatin and prominent nucleolus. **d** 100× CD20 positive. **e** 100× CD10 negative. **f** 100× Bcl 6 positive. **g** 100× Bcl2 positive. **h** 100× Ki-67 Proliferation Index was 50%

**Fig. 2:**
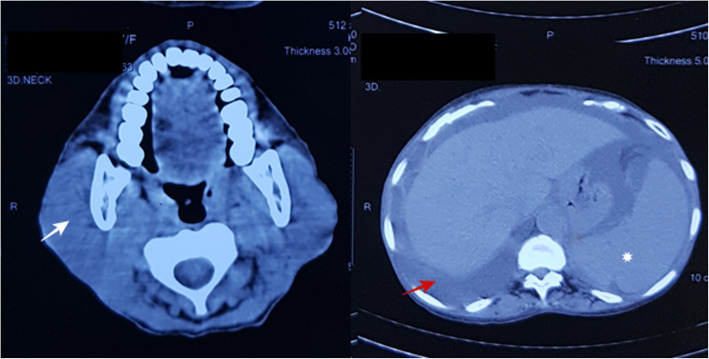
**a** NCCT neck showing a mass lesion in the region of parotid and level II with focal extension to paravertebral space and obliteration of parapharyngeal space (white arrow). **b** NCCT abdomen showing splenomegaly (star) and ascites (red arrow)

**Fig. 3:**
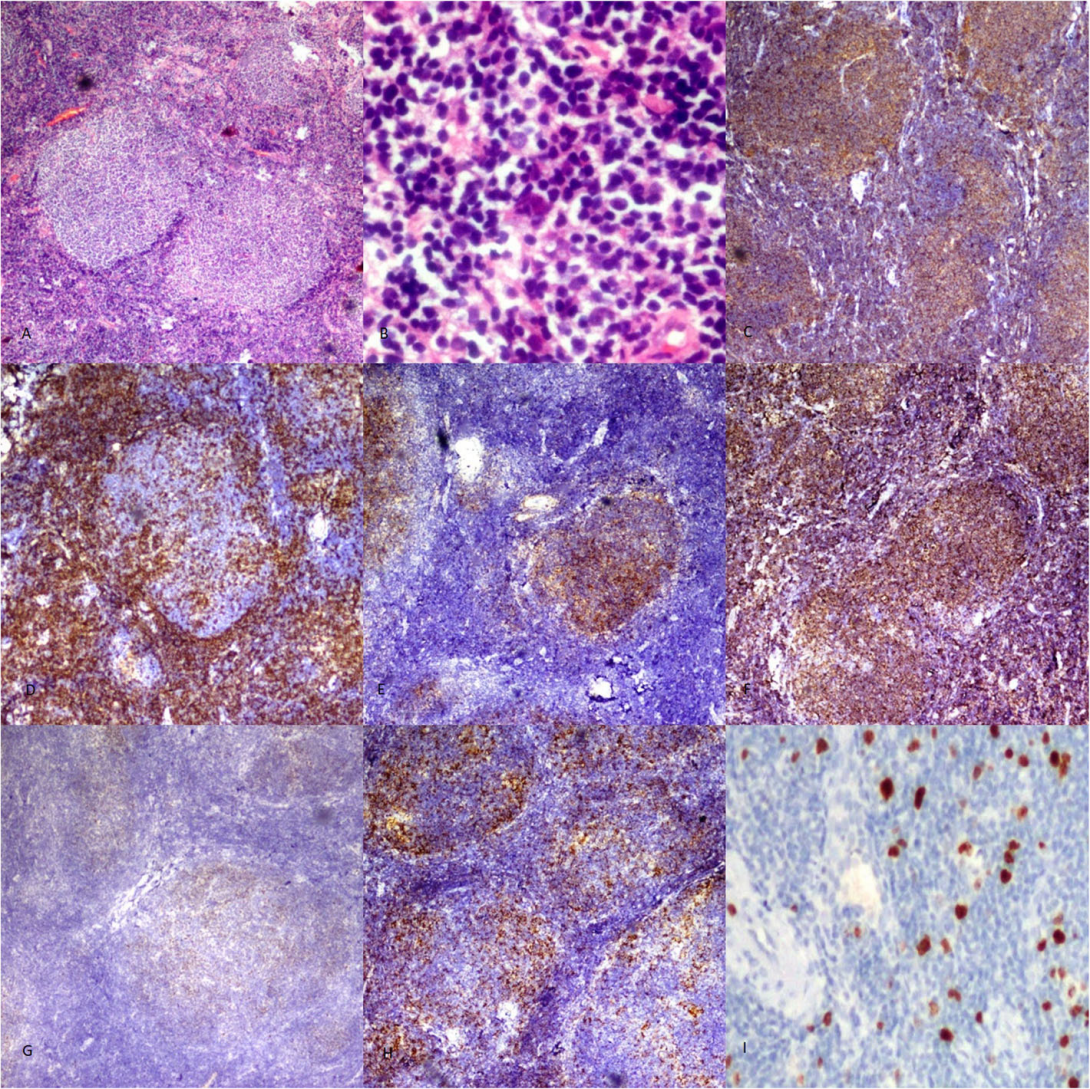
Histopathology images of biopsy specimen done in 2018 suggestive of follicular lymphoma. **a** 40× lymph node displaying uniform follicles arranged back to back. **b** 400× the atypical lymphoid cells show cleaved nucleus, scant cytoplasm. No centroblasts present. **c** 40× CD 20 positive. **d** 40× CD 3 negative. **e** 40× CD 10 positive in the follicular cells and faintly in the interfollicular region. **f** 40× Bcl2 positive. **g** 40× Bcl6 positive. h 100× CD 23 positive follicular dendritic cells in the stromal meshwork. **i** 100× Ki-67 Proliferation Index was 15 to 20%

In 2004, our patient who was then 39 years old was diagnosed with follicular lymphoma at another hospital. Details of staging were not available to us and could not be retrieved. She was prescribed prednisolone and chlorambucil for a period of 5 years till 2009. It is to be noted that this is not the standard of care of FL in a young patient; it may have been prescribed due to the financial constraints that the patient faced. We could not communicate with her previous oncologist about further details of the treatment. She presented to the Medical Oncology clinic at our Hospital in March 2015 with complaints of slowly progressing swelling in the neck on the left side for a duration of 1 year. It was associated with low-grade fever and weight loss for the last 3 months. No history of pain was noted. On examination, she had right parotid swelling measuring 6 × 5 cm with multiple right-sided lymph nodes in levels Ib–IV. A biopsy was done from the cervical lymph node which showed effaced lymph node architecture. The neoplastic lymphocytes were large, had high nucleocytoplasmic (N to C) ratio, vesicular nuclear chromatin and few displaying prominent nucleoli. Mitoses were frequent with atypical ones. No necrosis or granulomas were noted. Immunohistochemistry (IHC) performed revealed these large cells to be positive for LCA, CD20, bcl6, Mum 1 and bcl2 with KI 67 index of 50%. The cells were negative for CD3 and CD10. According to the Hans algorithm [8], it was categorised as activated B cell type (ABC) of DLBCL (Fig. 1a–h). Her CBC, LFT, SE, RFT and serum LDH were within normal limits except for a haemoglobin of 10.2 g/dl. A contrast enhanced computed tomography (CECT) of the neck, chest, abdomen and pelvis was done which revealed, in addition to the parotid swelling and cervical lymph nodes, multiple enlarged inguinal nodes on the left side, largest 3 × 2 cm, and a heterogeneously enhancing mass in the uterine cervix measuring 5.3 × 7.5 × 5.6 cm. Biopsy of the lesion from the uterine cervix showed similar findings as observed in the lymph node. The cells were large, arranged in diffuse sheets and displayed similar immunomorphology and were diagnosed as DLBCL, ABC type. A bone marrow aspiration and biopsy was done, and it showed only normal marrow elements. She was thus staged as diffuse large B cell lymphoma, activated B cell subtype stage IIIE (Cotswold modification of Ann Arbor staging [9]) with International Prognostic Index (IPI) [10] score of 2/5. She was treated with 6 cycles of CHOP chemotherapy regimen (cyclophosphamide 750 mg/m^2^, doxorubicin 50 mg/m^2^ and vincristine 1.4 mg/m^2^ on day 1 and prednisolone 100 mg on days 1–5; cycles repeated every 21 days). She tolerated chemotherapy without any serious adverse events. She had very good response to chemotherapy with a decrease in size of all cervical and inguinal lymph nodes as well as the parotid swelling. The patient was advised a CECT neck/chest/abdomen/pelvis for response; but she defaulted and was lost to follow-up. Again, she presented in April 2018 after a gap of 3 years with history of painless progressive swelling in the right side of the neck for the last 1 year. On examination, the patient was in good general condition. Multiple cervical groups of lymph nodes in levels Ib–IV on the right side were enlarged, largest 5 × 4 cm. They were firm in consistency, fixed to underlying structures without skin involvement and non-tender on palpation. Abdomen was distended, and shifting dullness could be elicited suggestive of free fluid. No hepatosplenomegaly was noted. The rest of systemic examination were within normal limits. CBC showed reduced haemoglobin of 9.4 g/dl. LFT and serum LDH was within normal limits. Serum creatinine and uric acid were elevated (1.9 mg/dl and 7.8 mg/dl). In view of deranged serum creatinine, the patient underwent a non-contrast CT of the neck/chest/abdomen/pelvis which revealed a conglomerate lymph nodal mass in the right cervical region measuring 5.5 × 4.4 × 4.2 cm involving the parotid gland with multiple smaller lymph nodes, gross ascites and a bulky uterine cervix measuring 6.7 × 9 × 9.2 cm (Fig. 2a, b). Biopsies of the lymph node and cervix were taken. The lymph node was partially effaced with follicular architecture. The neoplastic lymphoid cells were small to medium with predominant centrocyte having cleaved nuclei. The centroblasts were 5 to 8/hpf. No diffuse areas were noted. The neoplastic follicles were positive for CD20, CD10, Bcl2 and Bcl6. FDC meshwork was highlighted by CD23. Ki 67 was 15 to 20%. The uterine cervix also showed similar IHC findings except that the follicular pattern was not well made out. The cells were diffusely arranged, and the Ki 67 was around 20%. The final diagnosis was follicular lymphoma grade 1/2 with follicular areas in the lymph node and follicular lymphoma grade 1/2 with diffuse areas in the uterine cervix (Fig. 3a–i). Cytology of ascitic fluid did not reveal any malignant cells. The bone marrow aspiration and biopsy revealed normal marrow elements without any evidence of infiltration by lymphoma. She was thus staged as follicular lymphoma stage IV, FLIPI – 2/5, grade 1/2. In view of significant ascites, she was planned for chemotherapy with CVP regimen (cyclophosphamide 750 mg/m^2^ and vincristine 1.4 mg/m^2^ on day 1 and prednisolone 100 mg on days 1–5; cycles repeated every 21 days). After 6 cycles of chemotherapy, she has good partial response and resolution of ascites on repeat imaging with CT. At last follow-up in February 2020 (18 months after chemotherapy), she has maintained the partial response. She is planned for three monthly follow-ups with history and examination. Repeat imaging will be done at signs of clinical progression or once a year whichever is earlier.

## Discussion

FL is an indolent NHL. It arises from germinal centre B cells, and its pathogenesis is incompletely understood. The hallmark is the t(14;18) resulting in bcl2 overexpression; however, it is seen only in about 85% of the cases [11]. Its natural history and prognosis is variable with some patients having waxing and waning painless lymphadenopathy over years while some patients present with rapidly growing lymph node masses or disseminated disease necessitating immediate treatment to relieve compressive symptoms or cytopenias [12, 13].

HT of FL refers to evolution of the low-grade lymphoma to a high-grade lymphoma usually, DLBCL. Incidence of HT is about 1 – 4 % per year [5–7]. Clinically patients present with B symptoms (Weight loss, fever, night sweats), rapidly progressive lymphadenopathy, cytopenias, extra nodal involvement, effusions and elevated LDH or hypercalcaemia. It usually portends a poor prognosis. Before the era of rituximab, median survival was around 2 years; however, in the current era, the median survival of around 5 years has been reported [14, 15].

The exact pathobiology of HT is unknown. Two hypotheses are generally considered—divergent evolution of two distinct tumour populations from the original progenitor cell or emergence of an aggressive subclone from FL cells. Transformed FL usually retains the original t(14;18) in addition to additional abnormalities. Mutations of TP53 gene and bcl6 are noted in significant number of cases [16–18]. Kridel et al. evaluated samples of transformed FL by gene expression profiling and identified that about 80% transformed FL are germinal B cell type compared to 16% that are activated B cell type and about 24% of them were of double-hit type implying they had mutations in both bcl6 and c-myc genes. However, they could not predict an adverse outcome based on these features [19].

Re-biopsy of a node with suspected HT is the gold standard for diagnosis. The transformed lymphoma may have a germinal centre pattern, but with large cells effacing the follicular pattern. Risk factors for transformation include of higher FLIPI score at diagnosis and grade 3 FL [20]. PET-CT scanning may also aid in diagnosing transformation as transformed FL may have higher SUV uptake. PET-CT also aids in selecting lymph node groups for biopsy [21]. Presence of B symptoms, elevated LDH, beta-2-microglobulin or decreased albumin are also associated with higher risk of transformation [7, 14]. The use of maintenance rituximab may lead to lower rates of transformation [5].

The appropriate management of transformed FL is individualized. Casulo et al. formulated an algorithm for the management of transformed FL. They suggested the confirmation of HT with a biopsy from an FDG-avid lymph node group. In case of a young fit patient, they recommend an anthracycline-based chemotherapy regimen with rituximab, if previously not exposed, followed by consolidation with autologous stem cell transplant (ASCT) or salvage chemotherapy followed by ASCT, if previously exposed to anthracyclines. In older patients, if fit, they recommend considering a similar regimen as in young patients followed by consolidation with radioimmunotherapy or, if unfit, only palliative treatment with lenalidomide [22].

Our patient was initially treated with chlorambucil and prednisolone for 5 years. She was not exposed to anthracyclines or rituximab. She was offered treatment with R-CHOP chemotherapy regimen at transformation. However, in view of financial constraints, she received only 6 cycles of CHOP without rituximab. She had good clinical response; however, she defaulted without a follow-up scan to record the exact response to treatment. ASCT could not be planned as she defaulted. She then presented after 3 years with a diagnosis of follicular lymphoma. In the reported literature, most cases of transformed FL usually relapse with the higher-grade histology. Our case is unique in that at relapse, the histology was of the lower-grade FL.

Xu et al. described two cases of DLBCL which after successful treatment relapsed with FL. In one case, the diagnosis at relapse was pure FL, and in the second case, it was composite lymphoma with FL and DLBCL components [23]. This is a very rare event. We could not find any other cases in which a high-grade lymphoma relapses as follicular lymphoma after treatment. Our case is unlikely to be a case of composite lymphoma as two sites of disease were biopsied at two time points (2015 and 2018) and did not reveal different morphological subsets of disease, i.e., both FL and DLBCL.

A PET-CT should ideally have been done, and this would have helped us identify a lymph node group that would probably contain transformed FL. However, this is unlikely as two sites of disease were biopsied both at initial transformation and at relapse. The probable explanation for this phenomenon is that a subclone of cells transformed to DLBCL and this subset got eliminated with the anthracycline-based chemotherapy. This is a hypothesis derived from extrapolation of the work done by Casulo et al. [22]. It is to be noted that after 6 cycles of anthracycline-based chemotherapy in 2015 at the time of transformation, even though the patient had significant response, residual lymph nodes were palpable. A biopsy at this time point would have helped us know the exact kind malignant cells left after chemotherapy. The low-grade FL cells were probably not eliminated and thus may have contributed to relapse. This is understandable as DLBCL is usually considered a curable lymphoma and FL is not. This hypothesis can be confirmed by gene expression profiling of tissue samples of the biopsy specimen at diagnosis, transformation, post-treatment and relapse.

## Conclusions

We have presented a case of FL that has transformed to DLBCL after 10 years of diagnosis. After HT, she was treated with CHOP chemotherapy and the patient relapsed again after 3 years with follicular lymphoma histology. This case highlights the unique and varied natural history of follicular lymphoma that may be attributed to different subclones of malignant cells that may have arisen from a common progenitor FL cell and differential effect of chemotherapy on these subclones.

## Data Availability

Not applicable

## Abbreviations

DLBCL: Diffuse large B cell lymphoma
FL: Follicular lymphoma
NHL: Non-Hodgkin lymphoma
HT: Histologic transformation
T.: Tablet
LCA: Leukocyte common antigen
IHC: Immunohistochemistry
ABC: Activated B cell type
CECT: Contrast enhanced computed tomography
IPI: International Prognostic Index
ECOG PS: Eastern Cooperative Oncology Group Performance Status
CBC: Complete blood count
LFT: Liver function tests
RFT: Renal function tests
SE: Serum electrolytes
LDH: Lactate dehydrogenase
CT: Computed tomography
FDC: Follicular dendritic cell
FLIPI: Follicular Lymphoma International Prognostic Index
PET-CT: Positron emission tomography–computed tomography
SUV: Standardised uptake value
ASCT: Autologous stem cell transplant
